# The application of texture quantification in hepatocellular carcinoma using CT and MRI: a review of perspectives and challenges

**DOI:** 10.1186/s40644-020-00341-y

**Published:** 2020-09-22

**Authors:** Ismail Bilal Masokano, Wenguang Liu, Simin Xie, Dama Faniriantsoa Henrio Marcellin, Yigang Pei, Wenzheng Li

**Affiliations:** 1 Department of Radiology, Xiangya Hospital, Central South University, Changsha, 410008 Hunan China; 2 Department of Anatomy and Neurobiology, School of Basic Medical Sciences, Central South University, Changsha, 410013 China

**Keywords:** Hepatocellular carcinoma (HCC), Texture quantification, Radiomics, Clinical validation

## Abstract

Recently, radiomic texture quantification of tumors has received much attention from radiologists, scientists, and stakeholders because several results have shown the feasibility of using the technique to diagnose and manage oncological conditions. In patients with hepatocellular carcinoma, radiomics has been applied in all stages of tumor evaluation, including diagnosis and characterization of the genotypic behavior of the tumor, monitoring of treatment responses and prediction of various clinical endpoints. It is also useful in selecting suitable candidates for specific treatment strategies. However, the clinical validation of hepatocellular carcinoma radiomics is limited by challenges in imaging protocol and data acquisition parameters, challenges in segmentation techniques, dimensionality reduction, and modeling methods. Identification of the best segmentation and optimal modeling methods, as well as texture features most stable to imaging protocol variability would go a long way in harmonizing HCC radiomics for personalized patient care. This article reviews the process of HCC radiomics, its clinical applications, associated challenges, and current optimization strategies.

## Introduction

Hepatocellular carcinoma (HCC) is characterized by an increasing incidence, higher mortality, and morbidity burden. Currently, it is the second most common cause of cancer-related death worldwide, with about 50 % of all cases occurring in China alone [1]. The high mortality and morbidity burden mostly results from the late presentation of the disease in the majority of patients. In most cases, the diagnosis and follow-up of hepatocellular carcinoma, especially in the setting of cirrhosis can be effectively achieved using computed tomography (CT) and magnetic resonance (MR) imaging without the need for histological confirmation [2]. In recent years, there is an increasing need for better characterization of tumor heterogeneity and prediction of survival outcomes to permit individualized patient care. In this regard, the conventional qualitative CT and MR imaging modalities have fallen short, and consequently, the growing demand for more objective quantification of texture features.

Quantitative texture analysis of medical images – considered as a virtual biopsy technique – explores the microscopic details of a tumor by taking advantage of the spatial distribution and variation in the gray-level intensities of the pixels/voxels that make up the tumor images [3]. It has been expanded into the field of radiomics, which is an evolving research area that entails extracting large texture data to generate predictive models for prognostication, better tumor characterization, and assessing treatment responses. Although the Barcelona Clinic Liver Cancer (BCLC) is still the most popular treatment guideline, HCC radiomics analysis has proven useful in the guidance of treatment strategies and the prediction of therapy response. Despite its convenience and advantages, the full clinical application of radiomics analysis has been hampered by the absence of a standard execution guideline, delaying the clinical validation of the aforementioned applications. Promising studies on HCC applied radiomics differently in terms of the imaging protocol, segmentation methods, and model construction. However, for patients to fully benefit from a clinically validated radiomics, certain challenges about HCC texture quantification need to be addressed in the multi-step process of radiomics. This article explores the prospects and challenges in data acquisition, segmentation and modeling methods by reviewing recent developments in the clinical applications of HCC radiomics, which is vital for guiding its clinical applications to ensure a personalized medicine with optimized treatment strategies for better patient prognosis.

### Data acquisition and challenges

Most texture quantification of HCC is carried out using CT or MRI, as indicated in previous literature [4–6]. The universal problem associated with texture quantification in all available CT and MRI imaging modalities is in choosing the best imaging protocol, tumor segmentation methods, stable texture parameters, and the radiomic software tools used for analysis. In clinical work, different authors use different imaging parameters, depending on the equipment available in their institutions, which can account for the heterogeneity in tumor quantification [7, 8]. However, not all imaging parameters significantly affect the robustness of texture features; some vital parameters such as variations in scanner model, contrast injection rates, pixel resolutions, signal-to-noise ratio (SNR), and reconstruction algorithms have an obvious influence on the quantification of HCC texture feature. Therefore, it is important to develop a multi-parameter model that automatically corrects the variation in the various key data-acquisition parameters, or establish a comprehensive algorithm with a controlled imaging protocol that can provide stable texture features and enable an objective comparison between HCC radiomics studies, thereby promoting its clinical validation.

#### CT-based radiomics

In general, CT-based radiomics studies have shown that the variation of image acquisition parameters such as slice thickness, reconstruction algorithms, image resolution, contrast medium, and scanner type has the most significant influence on texture quantification [9–11]. In particular, reconstruction algorithms, pixel resolution, changes in contrast injection rates, and scanner models have been specifically implicated in HCC radiomics [9, 10, 12]. Regarding slice thickness, thinner image slices (1.25 and 2.5 mm) yield more quantitative texture information than thick slices (5 mm) [11]. Furthermore, the reconstruction algorithms: adaptive statistical iterative reconstruction (ASIR), model-based iterative reconstruction (MBIR) and the filtered back-projection (FBP) have each been shown to affect the quantification of certain texture features of liver lesions (*p* < 0.002) [10]. And in patients who had a retest contrast-enhanced CT scan 2 weeks apart, Perrin et al. showed that the variations in pixel resolutions and contrast injection rates could affect the number of reproducible texture features (gray-level co-occurrence matrix, GLCM; gray-level run-length matrix, GLRLM; intensity histogram and local binary patterns) between test and retest scans (concordance correlation coefficient of > 0.9) [9]. Also, each scanner model comes with a unique built-in pitch setting that can also influence the type of radiomics features extracted by the scanner. Finally, all other things being equal, the change in tube current and voltage [13], and the radiation dose [10] have no significant impact on HCC texture feature quantification.

#### MRI-based radiomics

In contrast to CT, determining the effect of data acquisition parameters on MR texture features can be more complicated because the imaging parameters, as well as contrast agents, bear no linear relationship with the signal intensity [14]. Generally, changes in echo time (TE), repetition time (TR), sampling bandwidth (SBW), spatial resolution, signal-to-noise ratio (SNR), field strength, scanner model, reconstruction algorithm, and parallel imaging acceleration factor, have been implicated in MRI-based radiomics [15–17]. Mayerhoefer et al. demonstrated that at a higher resolution, TE, TR, and SBW had a negligible effect on GLCM derived features in a phantom study using polystyrene spheres and an agar gel solution (PSAG) [15]. Generally, increasing spatial resolution and SNR improves the exploration of tumor heterogeneity [18]. In contrast, variation in the slice thickness of MR images doesn’t significantly affect the robustness of texture features [19, 20].

#### CT VS MRI-based radiomics

The superior diagnostic performance of MRI compared to CT in HCC has been documented [21] but their performance in radiomics analysis, however, has not been compared. As MRI has relatively higher sensitivity with a better spatial resolution and soft-tissue characterization, it might offer more robust texture features for tumor heterogeneity assessment than the CT [22]. A recent study compared the repeatability of CT and MR (using volumetric interpolation breath-hold examination (VIBE) and true fast MRI with steady state precession (TRUFISP) texture features of non-small cell lung cancer and showed 12 significant models that accurately predict overall survival but not tumor response. CT and MRI had a fairly similar predictive accuracy; 54.4% of CT texture features, 64.4% of TRUFISP and 52.6% of VIBE texture features were reproducible with a concordance correlation coefficient of ≥0.9 [23]. However as mentioned previously, simulation of the ground-truth textural composition of tissues of MR images can be more difficult, since the image signal intensities of tissues are strongly influenced by the MR acquisition parameters; moreover, images are more prone to artifacts that affect the quantitative analysis of texture features (especially the Gibbs ringing) compared to CT [14]. Thus, MRI-based radiomics signature may likely be more predictive of tumor heterogeneity but might be more susceptible to variations in imaging parameters compared to CT-based radiomics.

For both CT and MRI-based radiomics, good reproducibility of texture quantification is vital to the texture quantification. The reproducibility of the texture features is in turn affected by the imaging protocol and parameters.

Therefore, to improve the reproducibility of texture features, it is essential to understand how the alterations in the imaging protocol affect different texture features to allow the selection of suitable features under different imaging parameters (Table 1 below). This is because certain tumor texture features are less susceptible to even the most influencing parameter alterations. For example, some phantom studies [15–17] have demonstrated that GLCM-based features have greater reproducibility as they are more stable to variations in imaging parameters. Further studies using HCC phantoms are needed to determine if similar findings would be obtainable for hepatic malignancies.

**Table 1:** Studies that evaluated the impact of variation in imaging parameters on HCC texture quantification

Author	Study	Suitable Features extracted	Parameters	Parameter Variation	Impact on texture quantification	Conclusion
Perrin et al. [9]	CECT	254 features: GLCM, GLRLM, LBP, ACM, IH, FD	Contrast Injection rate (CIR)	Change in CIR 0.15 ml/s (range 0–2.5)	68/254features reproducible when variation CIR < 15% 50/254 features reproducible with variations of 50%	Quantification of features reduced as variability in CIR increases.
			Pixel resolution	Pixel resolution difference 7.27% (range 0–30.8%)	34/254 features reproducible with 15% variation in resolution. > 60 features reproducible with resolution variation < 5%	Quantification of features reduced as variability in pixel resolution increases
			Scanner model		75/254 features reproducible with same scanner and 35/254 with different scanner	Quantification of features reduced when > 1 scanner is used
Solomon et al. [10]	CECT	23 GLCM-features: Contrast, correlation, energy, homogeneity, entropy	Reconstruction algorithms:	Different reconstruction algorithm	Contrast: 32% lower with MBIR than with FBP	MBIR and ASIR significantly improved the quantification of texture features.
			MBIR, FBP and ASIR		Correlation: 37% higher with MBIR than FBP Energy: not significantly affected by algorithm	Radiation dose had no significant effect on texture features
			Radiation dose		Homogeneity: 15% higher with MBIR than FBP Entropy: unaffected No significant impact on texture features	
Mayerhoefer et al. [15]	3 T MRI	GLCM, GLRLM, IH, ARM, WAV based features	NA, TR, TE, SBW and pixel resolution	NA, TR, TE, SBW and pixel resolution at different values	Clinical resolution (MTX = 32 X 32; pixel size = 0.88 mm^2^): GLCM and GLRLM more sensitive to changes in NA, TR, SBW, TE than IH, ARM and WAV. Lower resolution: Sensitivity of all features to NA, TR, TE and SBW reduced	GLCM derived features were most robust to variations

*CIR* contrast injection rate, *GLCM* gray-level co-occurrence matrix, *GLRLM* gray-level run-length matrix, *LBP* local binary pattern, *ACM* angular co-occurrence matrix, *IH* intensity histogram, *FD* fractal dimension, *ARM* autoregressive model, *WAV* wavelet transform, *MTX* matrix size, *MBIR* model-based iterative reconstruction, *FBP* filter back projection, *ASIR* adaptive statistical iterative reconstruction, *NA* number of acquisitions, *TR* repetition time, *TE* echo time, *SBW* sampling bandwidth

### The process of HCC radiomics

**Fig. 1:**
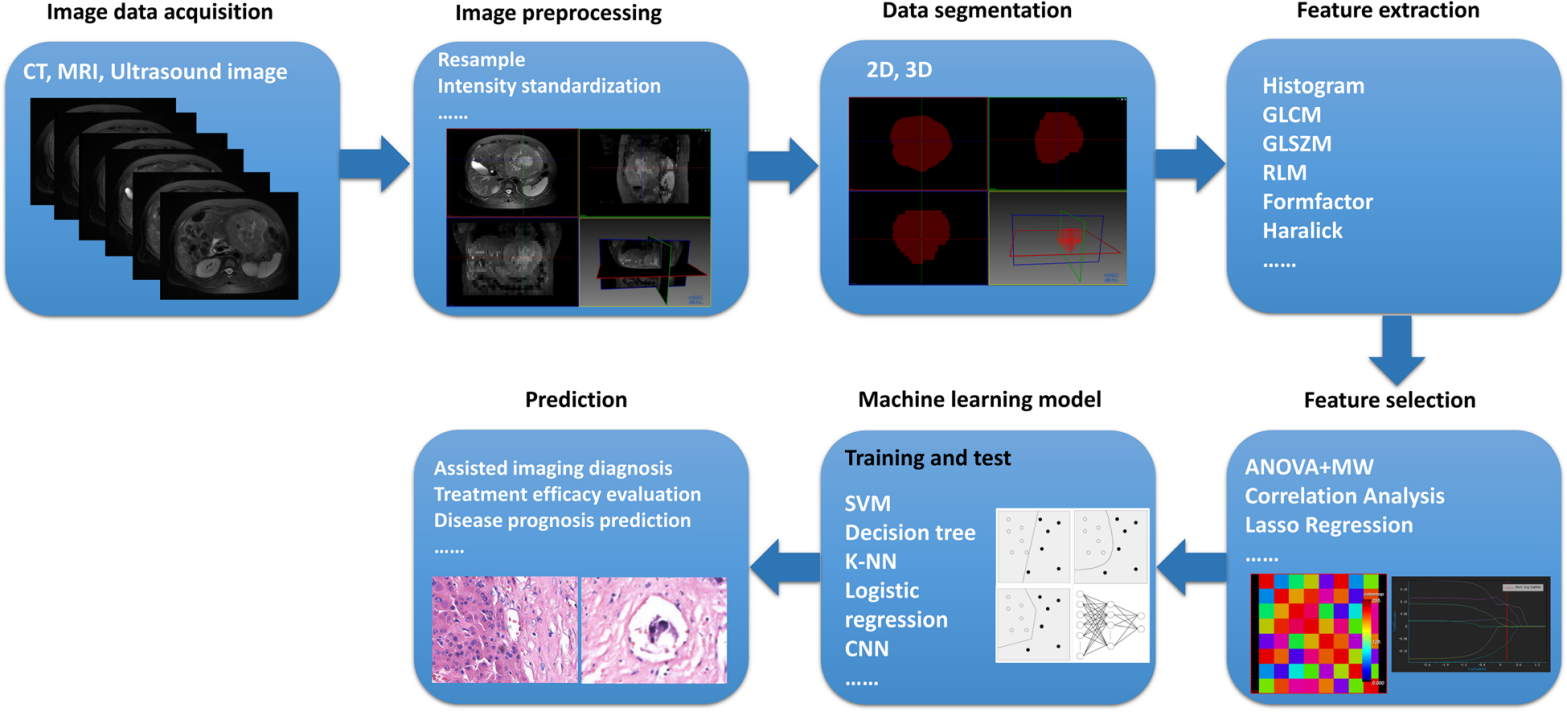
schematic diagram summarizing the steps in HCC radiomics

Texture quantification has been outlined in length in various studies [24–26]. Various software tools and statistical algorithms could be used on extracted image features to interpret tumor characteristics objectively and to determine the relationship between the tumor surface and the surrounding parenchyma [27]. A vast amount of texture features (currently around 50–5000) [28] from tumors can be converted into a mineable data from which radiomics and delta-radiomics signatures can be developed and then utilized for individualized patient care [29]. The process of HCC radiomics analysis begins with segmentation followed by feature selection, construction of radiomics signature, model building, and validation [4, 6] (Fig. 1).

#### Segmentation

The segmentation of the whole or part of a tumor using a radiomic software on a delineated region or volume of interest (ROI/VOI) for extraction is the first step in analyzing the texture of an image. The extracted features are described as semantic and quantitative. Segmentation can be manual, semiautomatic, or automatic. However, the semiautomatic segmentation method is currently more acceptable because it is less associated with bias from intra- or inter-observer variability compared with the manual delineation [8, 30] and less complex than the automatic method [31]. A study comparing the inter-observer variability of manual and semiautomatic segmentation showed that the semiautomated algorithm showed less variability compared with manual delineations (an interclass coefficient of 0.856 vs 0.776) [30]. The automated algorithms are limited by computational complexity, heterogeneity in HCC sizes and shape, and the need for a large amount of data set [30].

Current semiautomated segmentation algorithms proposed in the literature are broadly categorized into two: image intensity and tumor border-based methods [14]. The contour-based algorithms utilize the tumor contour detail while the intensity-based algorithms employ the tumor-parenchyma intensity gradient difference to execute segmentation. Algorithms that use tumor boundary information or intensity differences to segment tumors include region-growing (like the GrowCut), GraphCut, watersheds, livewires and active contours. GrowCut and GraphCut have become popular methods for semiautomated segmentation. Table 2 below summarizes the semiautomated segmentation algorithms.

**Table 2:** common semiautomated segmentation algorithms used in HCC

Segmentation	Algorithm	Description	Performance	Setback
Image intensity based [8, 32, 33]	Region growing e.g. GrowCut	Uses region-growing seed points to segment a tumor	Fast, low computational complexity, good reproducibility strong correlation with macroscopic tumor diameter	Segmentation errors due to boundary leakages, unsuitable for highly heterogenous tumors
	GraphCut	Constructs an image-graph of voxels connected by weighted edges	Can deal with tumors with odd shapes and mosaic intensity	Over segmentation or undesired ROIs when there are artefacts
	Water shed transformation	Segments tumor from parenchyma based on difference in gray scale intensity	Global segmentation	Over segmentation sensitive to poor tumor margins
Contour-based approach [34, 35]	Active contours, level-set and Live wires	Iteratively marks tumor contour from starting points on tumor edge	Faster than region growing methods	Rely on good initialization points and speed functions, sensitive to noise and poor tumor margins

##### Semantic features

Semantic features are the “apparent” tumor radiological features observed in daily practice. They include tumor location, size, and shape; enhancement characteristics, effusions, etc. [36]. Segal et al. used 28 semantic CT texture features to decode 78% of the genes expressed in liver cancers [37]. Similarly, two other studies used semantic features including liver-tumor interface difference and hypointense halo to develop a radiomic signature, which to some extent predicted microvascular invasion (MVI) of HCC [38, 39]. Although less affected by variations in imaging protocol, the semantic features suffer more subjectivity and variability, thus limiting their robustness [40]. However, they are still very useful, especially when combined with the quantitative features [41, 42].

##### Quantitative features

Quantitative features are those tumor characteristics that cannot be seen by mere observation of images; they require deciphering using various approaches including statistical-, model-, transform-based and structural methods [43, 44]. By and large, the statistical-based method is widely used in clinical practice for quantitative texture analysis. Quantitative features in the statistical model are grouped into first-order, second-order and higher-order statistics (Table 3), which have been used to represent the texture feature, and the second and higher-order statistics are of high importance for the evaluation of tumor characteristics in many HCC studies.

**Table 3:** The summary of the statistical model used in texture quantification

	Statistical Model		
	First-order	Second-order	Higher-order
Meaning	Frequency distribution of pixel/voxel gray-values without considering their spatial orientation [45].	Spatial distribution of pixel/voxel gray-levels in relation to their relative positions [46]	Characterizing images based on a unique interaction between the pixels/voxels that constitute the image [25].
Computation method	Histogram from which several texture features can be derived	Texture features obtained from the joint probability distribution of neighboring pixels	Mathematical algorithms that evaluate pixel intensities in relation to their neighboring pixels
Examples	mean gray-level intensity, uniformity, entropy, standard deviation, skewness, kurtosis	GLCM, GLRLM	NGTDM, NSZM, wavelet, and Gabor transform

*GLCM* gray-level co-occurrence matrix, *GLRLM* gray-level run-length matrix, *NGTDM* neighborhood gray-tone difference matrix, *NSZM* neighborhood size zone matrix

#### Challenges in tumor segmentation

A big challenge in tumor segmentation is in choosing the part of the tumor to be used in mining the radiomic data. Different studies take a different approach to perform tumor segmentation; some authors use part of the tumor while others use the whole tumor. Furthermore, other authors included the tumor and peritumoral region, and recently, Blanc-Durand et al. segmented the entire liver to build a whole-liver radiomics [47]. Francesca et al. showed a texture analysis of the entire tumor to yield more feature information, because texture characteristics of a particular ROI in the tumor, just like a biopsy sample, may not be the true representation of the entire tumor heterogeneity [48]. While some authors advocated exclusive segmentation of the tumor [5], more recently, others extended into the adjacent peritumoral tissue to generate a combined intra and peritumoral ROI/VOI. The latter approach has been shown to provide more detail on tumor heterogeneity, especially about MVI status [29, 49, 50]. A recent study demonstrated that variations in the methods of tumor segmentation affected the quantification and robustness of tumor texture features and emphasized the need for adopting a segmentation method from which the most stable radiomics features could be extracted [8].

### Radiomic model

After segmentation of the ROI/VOI, appropriate texture features are selected to build, train, and validate a predictive radiomic model.

#### Feature selection

Because the number of texture features obtained from a tumor can be quite large, the purpose of the feature selection step is to select only clinically relevant texture features that would be incorporated into a radiomic model. Extracted features are often narrowed to not more than the sample size [51] to avoid the curse of dimensionality, which occurs when the model is over-fitted with so many features: including redundant and irrelevant features. This affects the model’s performance on a new dataset due to high variance, leading to erroneous predictions [52]. There are various feature selection methods [53] but the most commonly employed approach is the filtration technique that is used to remove noise from selected features by highlighting texture features of specified size on the spatial scaling factor (ranging between 2 and 6 mm) [54]. Relevant and stable filtered features are computed by statistical methods including intraclass correlation coefficient (ICC), Students t-test, Mann-Whitney U test, etc. [26].

#### Model building and training

**Fig. 2:**
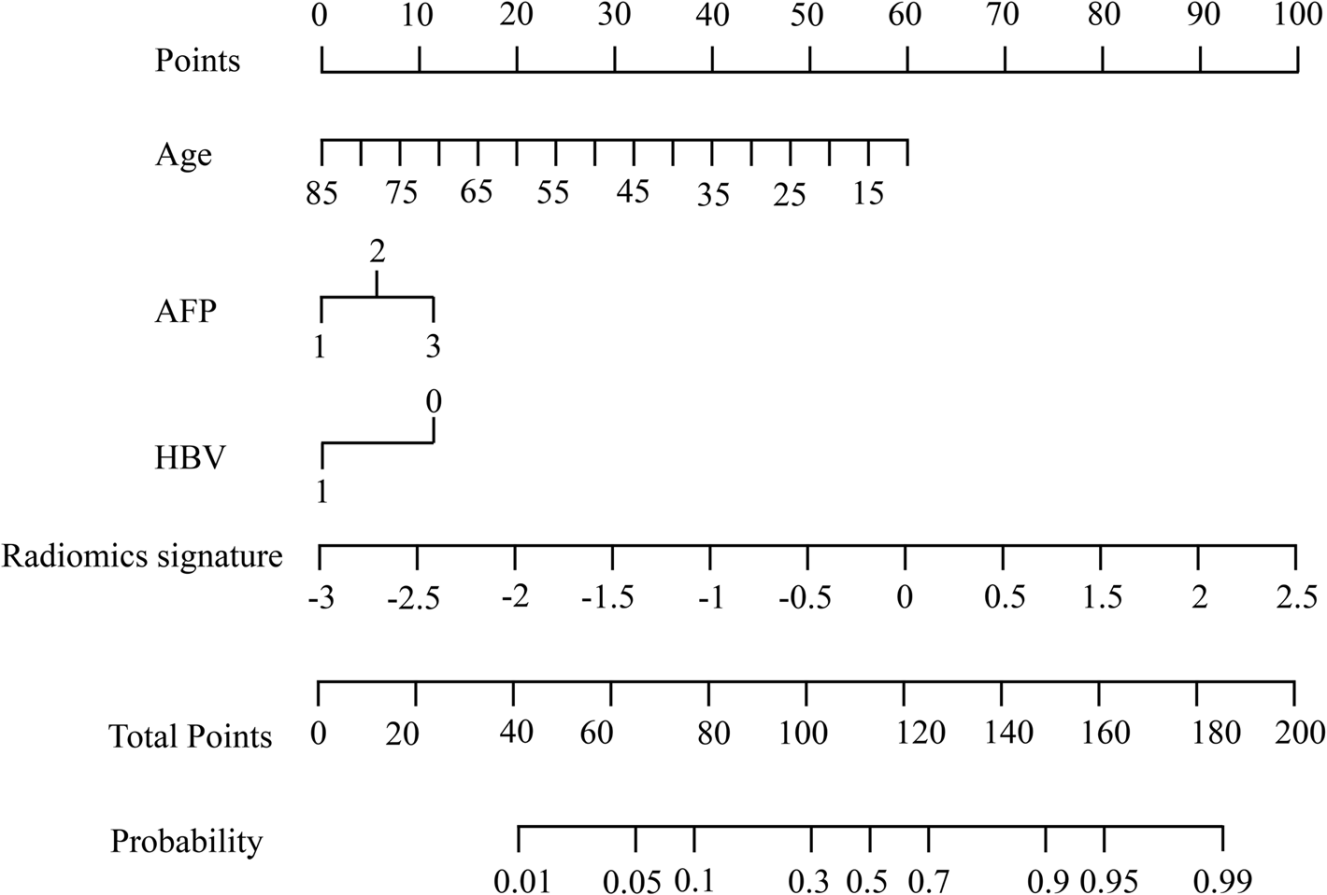
Illustration of a radiomic nomogram using clinical, laboratory and radiomics signature, AFP = alpha-fetoprotein, HBV = hepatitis B virus [56]

Since there are no established standard guidelines, researchers use different methods to build predictive models; however, regardless of the modeling method applied, the technique should be fully documented in a pellucid and easily reproducible manner [24]. Modeling can generally be supervised, semi-supervised or unsupervised. The commonly adopted method is the supervised approach in which a model is trained to characterize tumor heterogeneity or predict an outcome by feeding it with specific ground truth clinical labels and then tested for its performance [26]. If all the different models had the same predictive performance, comparison between studies would have been easier; however, because different modeling methods have different predictive accuracies, there is a need to identify the model with the best performance to achieve the desired clinical endpoint. On this regard, Ni et al. demonstrated that a combination of the LASSO+GBDT models (least absolute shrinkage and selection operator plus gradient boosting decision tree) had a higher diagnostic accuracy (highest area under the curve) for MVI of HCC compared to the back-propagation neural network (BPNet), K nearest neighbors (KNN), support vector machine (SVM), random forest (RF), decision tree (DT), and Bayesian models [55]. Moreover, recent studies on HCC quantitative analysis have shown that a combined radiomic nomogram composed of clinical, laboratory, semantic, and radiomic signatures, has better predictive power than a single radiomic model (Fig. 2). This is because variables such as the alpha-fetoprotein levels (AFP level), Child-Pugh score, and HBV status influence tumor heterogeneity [42]. For example, Kim and colleagues [42] improved the performance of their radiomic model in predicting survival form a hazard ratio of 7.42 to 19.88 (*p* < 0.0001) by incorporating alpha-fetoprotein (AFP) levels, liver function status (Child-Pugh score) and tumor size. Similarly, high AFP (> 400 ng/ml) and AST (> 40 U/L) levels are two clinical parameters that independently predicted MVI of HCC with an accuracy of 72.4 and 65.5%, respectively. When combined with radiomic signatures, the accuracy of the combined model improved to 82.8% (AUC of 0.889) [29]. Another study showed that both clinical (consisting of patients age, AFP, HBsAg, and tumor size) and radiomic models performed poorly in predicting MVI (AUC of 0.734 and 0.783, respectively). But when combined together, the performance of the clinical-radiomic significantly improved (to AUC of 0.835) [56].

#### Validation

After generating a suitable model, the final step is to validate its capability in accurately predicting the desired clinical outcome for which it is being built. Models can be validated internally by either using split or cross-validation methods, or more preferably externally using independent patient cohorts not included in the model construction. Several statistical methods are used to validate a model’s performance including the concordance index (C-index) and time-dependent receiver operating characteristic curve (ROC), which are generally used for models built to predict survival outcomes. For models built to predict a particular event such as MVI; the area under the curve (AUC), sensitivity, specificity, and calibration are utilized for validation [26] (summarized in Table 4 below). The best model is the combined model (clinical-radiomic model) with the maximum AUC values in most studies and should be recommended in texture quantification analysis due to its highest performance.

**Table 4:** summary of studies showing the predictive performance of radiomics signature, clinical-radiological and the combined models

Study	Objectives	No. of subjects	The area under the ROC curve			Sensitivity			Specificity			Best model
			RS	CM	COM	RS	CM	COM	RS	CM	COM	
Ma et al. [56]	Preoperative prediction of MVI	157 (T:110, V: 47)	0.793	0.761	0.801	0.656	0.944	0.889	0.944	0.655	0.759	COM
Yang et al. [41]	Prediction of MVI	208 (T: 146, V: 62)	0.837	0.759	0.861	0.842	0.737	0.895	0.744	0.674	0.814	COM
Xu et al. [29]	Prediction of MVI and survival	495 (T:350, V:145)	0.806	N/A	0.889	0.755	0.653	0.898	0.719	0.760	0.792	COM
Zhang et al. [57]	Prediction of MVI	267 (T:194, V:73)	0.820	0.721	0.858	0.692	0.269	0.808	0.809	0.936	0.861	COM
Zhu et al. [58]	Prediction of MVI	142 (T:99, V:43)	0.773	N/A	0.794	0.750	N/A	0.812	0.815	N/A	0.852	COM
Zhang et al. [59]	Prediction of early recurrence	155 (T:108, V:47)	0.728	0.814	0.841	0.696	0.783	0.913	0.708	0.833	0.750	COM
Zhou et al. [60]	Prediction of early recurrence	215	0.817	0.781	0.708	0.794	0.784	0.824	0.699	0.619	0.708	COM

*T* training cohort, *V* validation cohort, *N/A* not available, *ROC* receiver operating characteristic curve, *RS* Radiomics signature, *CM* Clinical model, *COM* Combined model

#### Challenges

The performance of models varies because each modeling technique has its unique limitations. Thus, the main challenge associated with modeling is the dare need for selecting an appropriate method for a particular event to be predicted [55]. Notably, the supervised learning models often require large amounts of training variables, which sometimes may not be enough to achieve optimal training to permit selection of the most relevant features. For this reason, a semi-supervised modeling method can be used to respond to insufficient data labels [26].

### Clinical application

Texture analysis of CT and MR images using tumor alone, a combination of the tumor and peritumoral region, or the whole liver (including all lesions) to generate a single radiomics signature or a combined clinical-pathologic-radiomic model has been applied to characterize and grade HCCs, assess MVI, monitor treatment response, and predict clinical endpoints.

#### Tumor characterization

Texture quantification of CT and MR images has proven quite useful in the characterization of liver lesions and particularly in atypical HCC in the non-cirrhotic liver where the diagnosis can be challenging even with the liver-specific contrast-enhanced MRI [61]. Stocker et al. used GLRLM, GLCM and gray-level histogram derived features to differentiate between HCCs and benign lesions in non-cirrhotic livers [12]. Texture features from spectral attenuated inversion recovery (SPAIR) T2W images have also been used to differentiate HCC from hepatic hemangioma and liver metastasis [62]. Also, a combined clinical and radiomic model has been used to predict MVI in HCC [58]. Moreover, some authors have used texture analysis to assign specific treatment options to patients based on their tumor textural characteristics [63, 64].

#### Response assessment

Texture quantification has been successfully used in monitoring response after both surgical and locoregional treatment of HCC. By quantifying change in tumor heterogeneity, radiomics analysis has the potential of assessing HCC’s response to treatments; however, only a few studies explored this potential [46]. Kloth et al. compared CT texture analysis with modified response evaluation criteria in solid tumors (mRECIST) and perfusion CT in assessing tumor response after drug eluting-bead transarterial chemoembolization (DEB-TACE) and showed that texture quantification could augment perfusion CT and mRECIST in monitoring treatment response [65]. Additionally, Yu JY et al. compared the texture features of HCC before and after TACE combined with high intensity focused ultrasound (HIFU) therapy and concluded that skewness (AUC 0.76, *p* < 0.05) and entropy (AUC 0.736, *p* < 0.05) from arterial phase images a week after treatment were strong predictors of early response [66].

#### Prediction of survival

Texture analysis of HCC has been used to predict the survival outcomes of patients after various treatment strategies [47, 63, 67, 68]. A study showed that texture characteristics could be used to select patients for combined TACE plus sorafenib, as well as to predict survival; Gabor-1-90 (filter 0) and wavelet-3-D (filter 1.0) from portal phase CT images were predictive of time to progression (TTP) and overall survival (OS), respectively. They proposed that patients having lower Gabor-1-90 (filter 0) and wavelet-3-D (filter 1.0) would benefit from TACE plus sorafenib [63]. Similarly, preoperative texture features from MR images have been used to predict early recurrence of HCC after hepatectomy. The study demonstrated that entropy and uniformity from the arterial phase images were independent predictors of early recurrence in patients with tumors ≤3 cm (*p* = 0.031 and *p* = 0.014, respectively) while entropy and skewness from same phase images were independent predictors of early recurrence in tumors > 3 cm [22]. And to predict response after chemotherapy with sorafenib, entropy from CT portal venous phase images was significantly (*p* < 0.05) associated with overall survival in both training and validation cohorts [68].

Some of the vital clinical applications of HCC radiomics are summarized in Table 5 below, and the most robust texture features are obtained from the second and higher-order statistics, which are GLCM, GLRLM, Gabor, and wavelet transform.

**Table 5:** Summary of the studies on radiomics analysis of HCC

Authors	Objectives	Study	Significant features/model	Phase	Summary
Oh et al. [69]	Predict tumor grade and DFS	CECT	SD, MPP and skewness	AP	AP based CCR model correlated well with tumor grade and DFS after resection
S. Song et al. [70]	Differentiate hypervascular lesions	CECT	Histogram, GLCM and GLRLM	AP	AP features characterized hypervascular liver lesions
Mokrane et al. [71]	Verify indeterminate liver nodules	CECT	Radiomic signature using KNN, SVM, and RF	AP and PVP	Machine-learning-identified feature diagnosed HCC in patients with indeterminate liver nodules
Huang et al. [72]	Characterization of HCC based on gene expression	Gd-EOB-DTPA MRI	GLCM, GLRLM and GLSZM-based signature computed using SVM	AP, PVP, DP, and HBP	A radiomic model predicted DPHCC preoperatively
Ma and Peng et al. [56]	Prediction of MVI	CECT	Radiomic signature computed with SVM and LASSO	PVP	CCR model was useful in preoperative and individualized prediction of MVI
Yang et al. [41]	Prediction of MVI	Gd-EOB-DTPA MRI	Radiomic signature computed with LASSO	HBP, T1W and HBP T1 map	HBP T1W and HBP T1 maps radiomic signature were independent predictors of MVI
Zhu et al. [58]	Preoperative prediction of MVI	MRI	Uniformity, CP, CS and LRLGLE in CCR	AP	CCR model predictive of MVI
Zhang et al. [59]	Prediction of ER	Gd-EOB-DTPA MRI	Histogram, GLCM, HGLRE in CCR computed with LASSO	T2W, AP, HBP	CCR had a better predictive ability of ER
Zhou et al. [60]	Prediction of ER	CECT	Histogram and GLCM radiomic signature computed with LASSO	AP, PVP	AP and PVP based CCR was a significant predictor of ER
Zhang et al. [22]	Prediction of ER	MRI	Uniformity, entropy, and skewness	AP	AP features were independent predictors of ER.
Brenet Defour et al. [73]	Prediction of OS	CECT	Skewness	PVP	Skewness associated with OS and useful for selecting best candidates for resection.
Zheng et al. [74]	Prediction of OS and TTR	CECT	GLCM radiomic signature computed with LASSO	AP	Low rad-score correlated with aggressive tumor phenotypes and predictive of postoperative outcome
Song et al. [75]	Prediction of RFS	MRI	Histogram, GRLM, GLCM, GLSZM based signature computed with LASSO	PVP	Preoperative estimation of RFS
Kim et al. [42]	Prediction of survival	CECT	Histogram, GLCM, GLSZM, and 2 shape-based features incorporated into CCR using LASSO	AP	A CCR nomogram performed better in survival prediction
Fu et al. [63]	Treatment and prediction of TTP and OS	CECT	Gabor filter and wavelet transform	PVP	Appropriate selection of HCC’s for TACE plus sorafenib
Kloth et al. [65]	Response assessment after TACE	CECT/pCT	Entropy, mean heterogeneity, uniformity, and skewness	AP/PVP	Significant correlation between texture features and pCT parameters in prediction of response

*AP* arterial phase, *PVP* portal venous phase, *CCR* combined clinical-radiologic/pathologic radiomic model, *LRLGLE* Long-run low gray-level emphasis, *CP* Cluster Prominence, *CS* ClusterShade, *HGLRE* High gray-level run emphasis, *GLN* gray-level run-length nonuniformity, *GLGCM* gray-level gradient co-occurrence matrix, *GWTF* Gabor wavelet transform texture, *OS* overall survival, *TTP* time to progression, *TTR* time to recurrence, *DFS* disease free survival, *PFS* progression free survival, *BCLC* Barcelona Clinic Liver Cancer, *ER* early recurrence, *TACE* transarterial chemoembolization, *RFS* recurrence free survival, *DPHCC* dual-phenotype hepatocellular carcinoma, *pCT* perfusion CT, *RF* random forest, *KNN* K-nearest neighbor, *SVM* support vector machine

#### Relationship between the radiomics features and Histopathologic correlates

In principle, tumor grading and immunotyping are determined by histopathological examination. Linking robust radiomics features with specific tumor histological markers will improve clinical decision-making without resorting to invasive procedures. But translating this correlation to tumor pathogenesis and outcome is difficult due to a large number of texture features and heterogeneity in HCC radiomics studies.

Zhou et al. linked MRI-based mean intensity and GLRLM-nonuniformity to histologic grades of HCC. Low-grade tumors (Edmondson-Steiner grade I and II) had significantly lower mean intensity and higher GLRLM-nonuniformity values compared with high-grade tumors (grade III and IV). Lower mean intensity of high-grade tumors in this study is caused by intratumoral necrosis when the tumors advance and outgrow their blood supply – equating to reduced contrast delivery. GLRLM-nonuniformity denotes heterogeneity in tumor cellularity, vascularity, and mesenchymal distribution; hence the higher values in more aggressive tumors [76]. High-grade HCCs had significantly higher MPP (pixels with intensity values greater than 0) and SD compared with low-grade tumors [69]. In this study, however, the higher intensity values (indicating more contrast uptake, i.e. more blood flow) is probably due to angiogenesis, which is necessary for rapidly growing tumors to meet nutrient and oxygen requirements [77]. Perhaps intensity features change with growth and advancement of the tumor – with higher intensity values in rapidly growing tumors and lower values when they outgrow their blood supply and subsequently develop necrosis.

### The current optimization strategies to realizing a standard HCC radiomics

Most studies on the texture quantification were conducted on brain, lung, or breast tumors. Similar studies on liver tumors are needed to assess further the robustness of texture features extracted from different types of HCC, as demonstrated by Perrin et al. [9]. Several scientific organizations are making efforts to standardize the processes of texture analysis to enable its full incorporation into clinical practice. Recently, the use of radiomics software tools that comply with IBSI’s standards (Imaging Biomarker Standardization Initiative), an initiative that seeks to standardize radiomics feature extraction, has been advocated [5]. Furthermore, radiomics analysis using a controlled imaging protocol will reduce the variability of extracted texture features [7]. To achieve the best evidence-based medical practice and enable a smooth clinical application of radiomics, a full disclosure of technical details and a clear description of the radiomic models and other processes of data analyses is necessary to enable external validation of results, to foster a better comparison of different study findings, and enable sharing of data as proposed by [24].

Our review focused on CT and MR-based HCC radiomics because most of the studies in HCC texture quantification were conducted using CT and MRI and the challenges associated with their radiomics process have been better identified and addressed compared to the few radiomics studies using other techniques such as multimodal ultrasound and positron emission tomography (PET). However, just like the BCLC algorithm, which incorporates tumor characteristics and the liver function status, studies using multimodal imaging techniques, such as the PET, to analyze both tumor and liver texture signatures are needed. This will help in developing a robust algorithm that will incorporate not only HCC radiomics, but also radiomics of the liver function.

## Conclusion

Numerous studies have demonstrated the application of texture quantification, especially when combined with clinical and pathological variables, in the management of various forms and stages of HCC. However, the clinical validation of HCC radiomics has been limited by a lack of standardization in image acquisition protocol and optimization of the radiomics analysis procedure. Recent pioneering studies have identified some robust tumor radiomics signatures that are most resistant to protocol variations. Thus, further studies on these robust signatures and the development of a multiparameter-model that automatically corrects the discrepancies in the most influential data acquisition parameters or a comprehensive algorithm using a controlled imaging protocol can help reduce the heterogeneity in the quantification of tumor texture. Finally, the radiomics signature of tumor and liver parenchyma maybe useful for simultaneously assessing both tumor heterogeneity and residual liver function. Perhaps with further work, such functional radiomic signatures would prove useful in establishing a standard HCC radiomic algorithm.

## Data Availability

Not applicable.

## Abbreviations

HCC: Hepatocellular carcinoma
CT: Computed tomography
MRI: Magnetic resonance imaging
BCLC: Barcelona Clinic Liver Cancer
SNR: Signal-to-noise ratio
GLCM: Gray-level co-occurrence matrix
GLRLM: Gray-level run-length matrix
TE: Echo time
TR: Repetition time
SBW: Sampling bandwidth
ROI: Region of interest
VOI: Volume of interest
MVI: Microvascular invasion
ICC: Intraclass correlation coefficient
ROC: Receiver operating characteristic curve
AUC: Area under the curve
